# Sexology, sexual development, and hormone treatments in Southern Europe and Latin America, c.1920–40

**DOI:** 10.1177/09526951231213028

**Published:** 2023-12-06

**Authors:** Chiara Beccalossi

**Affiliations:** 4547 University of Lincoln, UK

**Keywords:** biotypology, hormones, Latin America, sexology, Southern Europe

## Abstract

Displacing the physiological model that had held sway in 19th-century medical thinking, early 20th-century medical scientists working on hormones promoted a new understanding of the body, psychological reactions, and the sexual instinct, arguing that each were fundamentally malleable. Hormones came to be understood as the chemical messengers that regulated an individual's growth and sexual development, and sexologists interested in this area focused primarily on children and adolescents. Hormone research also promoted a view of the body in which ‘hermaphroditism’, homosexuality, and ‘sexual perversions’ such as masochism and sadism were attributed to anomalies in the internal secretions produced by the testes or the ovaries. This article focuses on Spanish, Italian, Argentinian, and Brazilian sexology shaped by endocrinological research in the interwar period. First, it shows the key role hormone treatments played in the historical development of sexology in Southern Europe and Latin America. Second, it looks at how sexologists employed hormone research to study human sexual development in the early stages of life, and how they set about ‘correcting’ what they viewed as ‘sexual anomalies’.

## Introduction

In recent decades, scholars have increasingly paid attention to how the biochemical notion of hormones and the consequent development of synthetic molecules for medical and commercial uses have radically modified traditional definitions of body, sex, and gender. Since the beginning of the 20th century, the body has come to be understood as chemically regulated, and sex as not a fixed entity, but instead malleable (Gaudillière, 2003, 2004; Fausto-Sterling, 2000; Oudshoorn, 1990, 1994; Preciado, 2008; Roberts, 2007). Reflecting on how hormone research has affected the history of medicine and gender at large, Jean-Paul Gaudillière has referred to the ‘molecularisation of gender and medicine’ and the ‘molecularisation of the sexes’ to describe the way in which so-called sex hormones and hormone treatments have changed how sex and gender have been perceived in medicine (Gaudillière, 2003: 57–80).^1^ Sociologist Nikolas Rose has likewise drawn attention to the ‘molecularization’ of 20th-century medicine, highlighting both how contemporary biomedicine envisages life at a molecular level, and the ‘“molecular” style of thought’ characteristic of 20th-century medicine (Rose, 2007: 5–7, 12).

It would be tempting to interpret the changes brought about by a new chemical understanding of the functioning of the body as a paradigm shift. If the philosopher Arnold Davidson famously spoke about an anatomical style of reasoning being replaced by a psychiatric one to explain the transformation in the understanding of sexuality in the 19th and early 20th centuries, we, drawing on Gaudillière and Rose, might talk about a new style of reasoning that emerged in the early 20th-century medical and biological sciences, namely, the ‘molecular style of thought’ (Gaudillière, 2004). While endocrinology meant a return to the body and, therefore, to Davidson's anatomical style of reasoning, this occurred within a new conceptual framework. For early 19th-century sexology, the ‘body’ meant anatomy, in that ‘sexual perversions’ were thought to be a disease of, for example, the reproductive organs or genitals (Davidson, 2001: 1–29). For 20th-century endocrinology, the ‘body’ was a chemical compound, with ‘sexual perversions’ judged to be hormonal dysfunctions, when too many or too few chemicals had been released into the bloodstream – a ‘humoral style of reasoning’ more than an anatomical one. Leaving aside the question of whether hormones brought about a paradigm shift or a return to a previous mode of thinking, it is clear that the understanding of hormones, and ‘sex hormones’ in particular, enabled scientists to remap the human body; sex and sexual differences; and, importantly, clinical interventions.

Endocrinological research has greatly broadened sexual knowledge as well. By the end of the 19th century, psychiatrists such as Richard von Krafft-Ebing had established a rich taxonomy of sexual behaviours that encompassed aspects from homosexuality and fetishism to sadism and necrophilia, while in the 1930s, endocrinologists such as Gregorio Marañón recognised that there were many human variations and created an equally rich taxonomy of ‘intersexual’ conditions. The burgeoning interwar literature on individuals with intersex variations threw into doubt the rigid division between male and female sexes, and was built around observations of sexual development. Sociologist and historian of health sciences Adele Clarke, analysing the history of reproductive science, shows that hormone research played a pivotal role in scientific thinking about sex differences in the first half of the 20th century. She argues that between 1925 and 1940, the US established its global dominance in the field thanks to, among other things, the financial support of the National Research Council's Committee for Research in Problems of Sex. According to Clarke, this dominance is exemplified by the publication of the important 1932 volume *Sex and Internal Secretions: A Survey of Recent Research*, edited by Edgar Allen (Clarke, 1998). While Clarke's important work establishes the central role of endocrinology, it also provides a universalist perspective on Western science. The basic principles of endocrinology were certainly shared among scientists across the globe, but endocrinological knowledge about sex and sex differences was reconfigured in different ways in different regions. In this article I aim to avoid generalisations, as endocrinological research, like any form of scientific knowledge, circulates through networks, and is not only historically but also geographically situated (Raj, 2013). By focusing on a selection of Argentinian, Brazilian, Italian, and Spanish emblematic case studies from scientists who used endocrinological and sexual knowledge, I will show a history that cannot claim global supremacy, but which was nevertheless global. Central to my analysis is biotypology, a medical classificatory science that employed endocrinological research to study the body and emerged at the crossroads of endocrinology, sexology, and eugenics in interwar Italy. Established by the endocrinologist Nicola Pende, biotypology circulated widely in Southern European and Latin American scientific circles and within various public health organisations that helped governments implement eugenics programmes.

Historians working on Southern Europe and Latin America have explored the relation between so-called Latin eugenics and biotypology, demonstrating that the former was fundamentally based on the latter (Cassata, 2006; Turda and Gillette, 2014; Vallejo and Miranda, 2004). They have examined the extent to which Latin eugenics contributed to shaping sexual knowledge, which in turn influenced demographic campaigns in the interwar period (Mantovani, 2004; Miranda, 2003, 2011). While these studies have deepened our understanding of the history of eugenics in general, showing that Latin eugenics had specific characteristics that set it apart from Nordic eugenics and that Latin eugenicists shaped ideas of sexuality, they have ignored the central role of hormone research. This is surprising, as biotypology, which is central to Latin eugenics, is fundamentally medical knowledge based on hormone research. Some research has concentrated on the history of hormones in the geographical areas that are the focus of this article (Cepada and Rustoyburu, 2014; Rohden, 2001, 2008), but, remarkably, this strand of research has overlooked the role played by biotypology in popularising hormonal research in scientific circles, and how hormones became central to discourses within Latin eugenics on non-conforming sexualities and gender. While a few scholars have recently started to explore the role of hormonal research in Latin eugenics (Beccalossi, 2020; Lima, 2021; Rustoyburu, 2012), a full history of the hormone practices employed to normalise non-conforming sexualities and gender remains to be written. It is my hope that this article will be a contribution to this history.

In the article, I will show that biotypologists produced knowledge about bodies that did not conform to perceived normative standards of male and female morphology, and were part of a complex network of medical and biological discourses, clinical practices, legal discussions, and even eugenic concerns that varied between countries. Biotypology circulated through networks that embraced Latin eugenics and were therefore more visible in Southern Europe and Latin America than in Northern Europe. Argentina, Brazil, Italy, and Spain were not the only countries where biotypological thinking on sexual variations and sexuality was present: my choice to focus on these countries reflects my broader research interests in the history of biotypology. Argentina and Brazil were the two most scientifically advanced Latin American countries in the interwar period (Stepan, 1991: 15), and there was intense scientific exchange between Argentina, Brazil, Spain, and Italy (ibid.; Vallejo, 2004).

Looking at this history of sexology through the prism of the circulation of biotypological knowledge and practices helps us understand the global dimension of what we might call ‘Latin sexology’. This sexology took shape in countries that showed some degree of cultural affinity and where Latin eugenics circulated. I am interested here in what Laura Stoler calls ‘circuits of knowledge production’ (Stoler, 2001; see also Haynes, Fuechtner, and Jones, 2017). The global circuit of knowledge production I explore was also helped by political circumstances. Long-time fascist concerns with demographics, prolific populations, virility, and normalisation certainly facilitated the circulation of biotypological sexual knowledge within some countries. These concerns were shared by biotypologists. But it would be simplistic to credit fascism with all the success of biotypology. Some of the scientists I cover here were not fascist, most notably Marañón, a Republican who opposed the dictatorship of Miguel Primo de Rivera. Moreover, the historiography shows that groups across a wide political spectrum, from the far right to communists and even anarchists, shared eugenic concerns (Cleminson, 2000). While I attempt to place the emergence of biotypology in its political context, I focus on its intellectual history, demonstrating the contradictory nature of endocrinological ideas and practices developed by biotypologists.

In what follows, I will illustrate a global circuit of knowledge production about hormones and the clinical interventions that emanated from biotypology. By focusing on how biotypologists and endocrinologists analysed sexual development in Spain, Italy, Argentina, and Brazil, I will show that their sexual knowledge blurred the boundaries between the sexes. However, even though biotypologists and endocrinologists challenged ideas of binary sex, their actual practices aimed to restore binarism. Hormone treatments showed that sex and sexual characteristics were malleable, and hormone therapies were used to normalise individuals.

## The earliest stages of human development: Original bisexuality

In the second half of the 19th century, men of science increasingly endorsed the view that in the early phases of human development, the body was sexually undifferentiated, and while each individual would eventually develop the sexual characteristics of one sex, those of the other sex remained in a latent state. This belief was supported by the evolutionary theories and embryological observations of the time. In the enlarged 1927 edition of *Sexual Inversion*, British sexologist Havelock Ellis explained that the concept of a ‘latent organic bisexuality of each sex’, or what he called the theory of ‘embryonic hermaphroditism’, had been evident in ancient Greek philosophy since Plato, although it was not until the final decades of the 19th century that modern scientists such as Charles Darwin had been able to prove it (Ellis, 1927: 302–24).^2^ In *Variations of Animals and Plants Under Domestication* (1868), Darwin wrote that ‘in many, probably in all cases, the secondary characters of each sex lie dormant or latent in the opposite sex, ready to be evolved under peculiar circumstances’ (Darwin, 1868: 52).^3^ Although Darwin did not refer to ‘sexual perversions’ when writing about the original lack of sexual differentiation in human beings, the idea of a ‘latent organic bisexuality’ was explored at length in works on ‘sexual perversions’, and in particular on ‘sexual inversion’, which was a major concern in late 19th-century sexology.

Ellis provided a brief overview of those late 19th-century psychiatrists who employed the concept of latent organic bisexuality to explain certain non-reproductive sexual behaviours within an evolutionary framework. For example, American psychiatrists James Kiernan and G. Frank Lydston published a series of studies in the 1880s and 1890s in which they explicitly linked the phenomenon of ‘embryonic hermaphroditism’ to that of sexual inversion (Angelides, 2001: 40–1; Chauncey, 1982–3: 131–7). They argued that homosexuality was an ‘atavism’, a throwback to lower evolutionary forms (Herrn, 1995; Sulloway, 1979: 292–3). Ellis went on to review cursorily the findings of several European doctors, including Krafft-Ebing, who in the 1890s adopted the theory of original bisexuality to explain homosexuality. These psychiatrists associated homosexuality with the theory of degeneration; in their view, the original organic bisexuality of the body re-emerged in sexual inverts as their condition became tainted by degeneration. Thus, these psychiatrists couched their association between an original bisexuality, evolutionary theories, and sexual inversion in the rhetoric of pathology.

Other medical men, drawing more on embryology, tended to emphasise the natural and universal aspect of the original lack of sexual differentiation. Throughout the 19th century, embryologists and anatomists across Europe who analysed the earliest stages of the development of sex anatomy reported that the foetus in its earliest stages had no sex, or was fundamentally hermaphroditic. This led them to conclude that nobody was completely female or male (Brooks, 2012: 200–3). One of the most cited pieces of research within the field of sexology up until the 1930s – especially in Southern Europe and Latin America, which adopted embryology to understand sexual inversion – was published in 1884 by the French physiologist and endocrinologist Marcel Eugène Émile Gley. In his article ‘Les aberrations de l’instinct sexuel’ for the influential journal *Revue philosophique*, Gley observed that in the first weeks of gestation, the embryo was sexually undifferentiated, and therefore all human beings were potentially both male and female (Gley, 1884: 88–92). Eventually, the foetus developed in one direction, but the signs of an original anatomical bisexuality persisted. Using a theoretical framework still couched in the rhetoric of pathology and dominated by the sexual dimorphism that encouraged physicians to associate specific psychological traits with the male or female sex, Gley explained sexual inversion as a phenomenon whereby the body developed in one direction, say female, but the mind maintained the characteristics typical of the male sex.

By the end of the 19th century, medical researchers interested in sexual matters had accepted that the human embryo was originally neutral, with the implication that each individual carried the latent sexual characteristics of the opposite sex. Gley's thesis was adopted, for example, by French physician Julien Chavalier and, through Chavalier, by Krafft-Ebing and Freud, the latter observing that an original bisexuality was a universal phenomenon (Sulloway, 1979: 293–4 and n. 11). In Latin American countries such as Argentina and Brazil, European embryological and evolutionary theories were also employed to explain phenomena such as intersexuality and sexual inversion (Ben, 2000b; Ferla, 2004). The discovery of the chromosomal sex determination system at the beginning of the 20th century by North American geneticists Nettie Stevens and Edmund Beecher Wilson did not alter the endocrinologists’ view. They argued that chromosomes might set in motion the process of sex determination, but that hormones controlled sex differentiation.

In the first decade of the 20th century, endocrinologists already understood that hormones produced in the testes and ovaries controlled the development of secondary sexual characteristics and the genitals, and they took so-called ‘sex hormones’ to be the chemical messengers of maleness and femaleness. They initially believed that ‘female sex hormones’ could be found only in a female organism, and ‘male sex hormones’ in a male organism, with ovaries producing female sex hormones and testes producing male sex hormones. In this sense, endocrinology seemed to confirm earlier scientific anatomical research that held that the male and female organisms were fundamentally different. If, in the 19th century, the ovaries defined the essence of a woman and the testes that of a man, in the early 20th century hormones produced by the sexual glands defined both a woman and a man. Femininity and masculinity now depended on internal secretions. Endocrinologists seemed to confirm a dualistic concept of sex. Hormone replacement therapies were then developed to ‘correct’, for example, infertility in women and impotence in men, and even a lack of femininity in women or masculinity in men. The organotherapy developed by Charles-Edouard Brown-Séquard, which supposedly treated a lack of male sexual vigour and rejuvenated men, is well known (Oudshoorn, 1990: 165–6; Sengoopta, 2006: 36–8). Early endocrinological knowledge and treatments helped promote the ideal of a vigorous and muscular healthy man and a fertile and prolific healthy woman with pronounced secondary sexual characteristics. The androgynous man and woman were perceived as endocrinological anomalies who could be corrected through hormone therapies.

Despite endocrinology contributing to establishing normative masculinity and femininity, it also did much to disrupt the binary system of thinking about sex within the broader field of sexology, albeit with a number of contradictions. In the 1920s, the binary model of sex began to lose its dominant position as endocrinologists discovered that the ‘female sex hormones’ (what we now call oestrogens) were also produced by the testes and could be found in the urine of healthy men. In 1921, Viennese gynaecologist Ofried Fellner published an article on the growth of the uterus in female rabbits after treatment with testicular extracts. In 1927, Dutch biochemists announced that they had found female hormones in the testes of healthy men. Finally, in 1934, Bernard Sondeck found oestrogenic sex hormones, considered to be female, in the urine of stallions (Cleminson and Vázquez García, 2009: 154–5). By the 1930s, sex had been transformed into a concept of ‘relative sexual specificity’. Endocrinologists no longer believed in maleness and femaleness as two clearly defined and distinct endocrinal states; instead, they now subscribed to the idea that an individual's sex was the result of a balance of endocrinal factors. Healthy and normal bodies with testes could feature female hormones and some secondary sexual characteristics typical of the female sex, while healthy and normal bodies with ovaries could feature male hormones and typically male secondary sexual characteristics (Oudshoorn, 1990: 163–9).

Interwar genetic studies on those who had up to that point been called ‘hermaphrodites’ further challenged the binary understanding of sex. Alongside the study of hormones and their effects on secondary sexual characteristics, between 1916 and 1917 the German-born American geneticist Richard Benedict Goldschmidt published a couple of articles on what he called ‘intersexuality’ (Goldschmidt, 1916, 1917). Broadly speaking, the term indicated an individual who had characteristics of both the female and the male sex. Goldschmidt proposed that there were not only the male and female sexes. Instead, sex existed on a continuum, and between the male and the female sex there were individuals with sex variations resulting from factors such as genetics, endocrinology, physiology, and serology (Linge, 2021: 40–50). In medical literature, *intersexual* increasingly replaced the older term *hermaphrodite*, which was now considered inadequate because it focused on an individual's gonads (del Castillo *et al.*, 1944: 492). Studies on intersex variations not only undermined binary concepts of sex and complicated medical taxonomy, but also encouraged further studies on the role of hormones in the process of sexual differentiation. Endocrinologists came to identify multiple hormonal balances that produced a broad range of variation in bodies. Interwar endocrinological treatises and journals were filled with cases of individuals whose bodies did not conform to accepted standards of male and female morphology, and while they attempted to determine the ‘true’ sex of some individuals, they ultimately emphasised the existence of human variations.

Gregorio Marañón, a renowned Spanish endocrinologist who helped rethink how sexology viewed the sexes, provides an example of how, based on their work on intersex conditions, endocrinologists emphasised the variability of human bodies. Marañón studied sexual development in the context of his work on ‘intersexual conditions’. Among the most influential titles in his extensive body of work was *La evolución de la sexualidad y de los estados intersexuales*, published in 1930.^4^ Recognising that Darwin had already acknowledged that the male and female sexes were not opposing and antagonistic poles, Marañón emphasised that ‘the masculine and the feminine are not two diametrically opposed entities, but successive degrees in the development of a single function – sex’ (Marañón, 1932: 15). Between the two ideal sexes, which in reality did not exist, there existed ‘intermediate conditions’ (ibid.: 15–19).^5^ Studies in ‘sexual biology’ had demonstrated that ‘the “male-type” and the “female-type” are almost fantastic figures’, while the ‘conditions of sexual confusion – in a scale of infinite gradations which extends from flagrant hermaphroditism to forms so attenuated that they merge into normality itself – are so widely diffused that there is scarcely any human being whose sex is not tainted by a doubt, or at least the shadow of a doubt.… Full sexual differentiation is still rare… [t]he two sexes, masculine and feminine, are not two entities that stand opposed at all points’ (ibid.: 17). The sexual composition of an individual was always a mixture of male and female characteristics, with the predominant sex the result of a hormonal balance (Cleminson and Vázquez García, 2007: 122–78; Marañón, 1930).

Thus, Marañón suggested that between the predominant male type (male sex) and the predominant female sex, there was a spectrum of ‘intersexual conditions’, which encompassed homosexuals, hermaphrodites, ‘viriloid’ women, ‘feminiloid’ men, men with gynaecomastia, and so on. Hermaphroditism and homosexuality were part of the same phenomenon in Marañón's theory (Cleminson and Vázquez García, 2009: 155). This idea, which drew on the work of Alexander Lipschütz (1883–1980), a Chilean physiologist based at the University of Concepción, came to be known as the ‘theory of intersexuality’ (MacMillan, 2017). According to Marañón, the ‘infinite gradations’ of the sexes depended not only on the hormonal composition of each individual, but also on cycles of life development. Men and women both evolved from an original, undifferentiated state, but they followed different paths of sexual development. After adolescence, Marañón maintained, women went through a long period of ‘feminine maturity’, maternity, followed by a short ‘viriloid phase’, which ended with old age. For men, the infantile period was followed by a ‘feminoid’ phase, rudimentary and short, succeeded by a long virile period that extended into old age, during which they exhibited well-defined male secondary sexual characteristics (Marañón, 1932: 8). Men achieved a higher level of development, while women remained closer to an infantile, bisexual type. The woman was ‘an organism arrested, so far as her general evolution is concerned, in the borderland of adolescence by the necessity of specialising a large part of her activity in the transcendent function of maternity’ (ibid.: 82). Consequently, Marañón argued that men were made for physical and intellectual labour, while women were suited to maternity. Despite the fact that Marañón emphasised the variability of bodies and focused on intersex variations, he was not able to free himself from essentialist principles or the naturalisation of sexual differences. For example, he repeated many of the stereotypes favoured by biological determinists of his time: women were emotive, irrational, and linked to the world of feelings, while men possessed ‘a greater aptitude for abstract and creative functions’ (ibid.: 84–5).

## Nicola Pende's biotypology, sexual development, and moulding bodies

Marañón's work circulated widely in Southern Europe and Latin America. In his own time, he was recognised as an exponent of constitutional medicine. According to the Argentinian physician Arturo Rossi, Marañón was one of the most important figures of this branch of medicine, who established, among other things, an entire department devoted to the study of biotypology in his clinic in Madrid (Rossi, 1944: Vol. 1, 36). Marañón was interested in and adopted biotypology (Marañón, 1935, 1972[1926]), but he is not generally remembered by historians for this area of research.^6^ Biotypology was a medical classificatory science founded by the Italian endocrinologist Nicola Pende, who coined the term *biotipology* in 1922, from the Greek words *βίος* (life), *τύπος* (type), and *λόγος* (‘logos’, meaning word, study, or doctrine). Within a constitutional medicine framework, Pende devised a typology of the individual based on the study of combinations of different components of the body: physical constitution (weight, height, muscular mass, body shape), temperament (neuroendocrine system), and character (psychology). The underlying principle was that basic human types (biotypes) existed, which could be defined by different hormonal formulae. Each such hormonal body had a specific shape, as well as specific psychological characteristics and attitudes, and was prone to particular diseases. Dividing human constitutions into typologies was not new to constitutional medicine. The innovation in Pende's biotypology was the central role of hormones: the body became a hormonal body.

As defined by Pende, biotypology was the science of ‘the architecture and engineering of the individual human body’ (Pende, 1939: 1). Biotypology grew out of concerns typical of the interwar period. The destruction of national biological assets resulting from the First World War encouraged the development of Italian eugenics programmes that aimed to regenerate the national stock and increase the size of the population. Biotypology can be considered a form of eugenics, and historians have traditionally studied Pende for his role in shaping Italian eugenics (Cassata, 2006; Mantovani, 2004); only recently have they started looking at his endocrinological and sexological studies (Beccalossi, 2020). Pende belonged to the first Italian sexological association, the Società italiana per lo studio delle questioni sessuali, founded by Aldo Mieli in 1921, which also published the journal *Rassegna di studi sessuali*. In 1926, four years after coining the term *biotypology*, Pende established a centre exclusively devoted to his new discipline, the Biotypological Orthogenetic Institute in Genoa. This was a medical centre that focused on endocrinological research, and at the same time a eugenics and sexological institute. Pende's orthogenesis derived from the same premises as eugenics and had the same aim, the improvement of the race, although it differed through its advocacy of interventions after birth, so as not to infringe upon the tenets of the Catholic Church regarding every person's right to life.^7^ In practice, the institute aimed to improve the quality of the Italian stock using hormone treatments, and focused on the study and treatment of children and young adults (Gualco and Nardi, 1941: 163). The medical scientists working at the Biotypological Orthogenetic Institute offered premarital counselling and racially evaluated unions, favouring those that in the long term, they believed, would produce ‘fit’ offspring. They studied sexual dysfunctions, individuals with variations in sex characteristics, and to a lesser extent sexual variation. They also offered treatments for sexual dysfunctions such as impotence, and treated individuals with intersex conditions and even homosexuality (Beccalossi, 2020).

Like other men of science and sexologists before him, Pende believed that the earliest phase of each human being was sexually undifferentiated and that every human maintained a latent organic bisexuality. According to Pende, hormones contributed to the development of the secondary sexual characteristics of one sex. However, hormonal dysfunctions could at any time cause the re-emergence of the secondary sexual characteristics associated with the opposite sex (Pende, 1933: 30–1). In *Endocrinologia* (1923), Pende explained that in the early 1920s, endocrinologists were increasingly inclined to admit that the sexual glands were characterised by a ‘physiological hermaphroditism’. Indeed, according to studies from the period, there were ‘rudimentary male elements’ in the ovaries, while the testes also featured ‘female rudimentary elements’ (Pende, 1923: Vol. 1, 41). During ontogenesis, a dominant endocrine element (either the female or the male) took over, and the sexual characteristics of that dominant element developed throughout the organism due to internal secretions, while the characteristics of the other sex were inhibited and remained latent. In some pathological conditions, the internal secretions of the dominant endocrine elements decreased or halted, and the ‘rudimentary elements’ of the opposite sex, which until then had lain dormant in the genital glands, began developing, resulting in the appearance of the secondary sexual characteristics of the other sex (ibid.). According to other important endocrinologists such as Lipschütz, the Austrian Eugen Steinach, and the French-Russian Serge Voronoff, virilism in women and ‘feminilism’ in men emerged through this process (ibid.).

Pende's work exhibited a rich taxonomy of variations in sex characteristics. In *Endocrinologia*, Pende covered the ‘stati genitali’ (genital conditions) that were caused by dysfunctional genital glands. These conditions included ‘hermaphroditism’, ‘eunuchismo’, ‘eunucoidismo’, ‘virilismo’ in women, and ‘feminilismo eunucoide’ in men. According to Pende, sexual behaviours such as homosexuality or ‘erotism’ and ‘sexual perversions’ such as ‘sadism’ and ‘masochism’ were all ‘intimately connected to anomalies in the internal secretions of the genital glands’, and as such were considered ‘genital conditions’ (Pende, 1923: Vol. 2, 976).^8^ These non-reproductive sexual behaviours were ‘deviations’ in the sexual development of the body and ‘psychosexuality’ (ibid.: 976–1015). Thus, like Marañón, Pende conflated variations in sex characteristics such as intersexuality and homosexuality. Pende explained, however, that the sexual instinct was regulated not only by ‘sex hormones’, but by a complex interaction of all the hormones produced by the endocrine glands (ibid.: 1009).

In *Endocrinologia*, Pende did not describe the psychological characteristics of homosexuals. Instead, he confined himself to providing a physical description of each of the sexual variations he covered, seeming to take psychiatric studies for granted. Likewise, in his other works he only occasionally engaged in medical studies on the psychological characteristics of homosexuality. For example, in *Trattato di biotipologia umana individuale* (1939), he touched on the mental characteristics of sexual deviancies, repeating some of the stereotypes typical of psychiatric studies of the time: male homosexuals were not considered fit for the struggle for life, favouring, it was supposed, feminine occupations and displaying feminine tastes. Female homosexuals preferred male clothes, were aggressive, enjoyed sports, and had a propensity for typically male professions (Pende, 1939: 411). While fully aware of psychiatric studies on phenomena such as homosexuality, Pende was reluctant to stray into other medical disciplines. But he could not avoid crossing disciplinary boundaries. Indeed, by associating perceived deviations of human behaviour such as homosexuality with endocrinological anomalies, Pende was supplying a conceptual link between the behaviour of human beings and their bodies. Hormones were the link between the psychological and morphological spheres; Pende's theoretical apparatus thus linked psychiatry and anthropometry through endocrinology.

When covering ‘genital conditions’, Pende paid particular attention to the early phases and sexual development of humans. He explained that the internal secretions of the genital glands, what we now call testosterone and oestrogen, strongly influenced the development of secondary sexual characteristics, as experimental research with human and animal castrations and observations about ‘eunuchs’ testified (Pende, 1923: Vol. 1, 39). In works such as *Endocrinologia* and *Anomalie della crescienza fisica e psichica* (1929), Pende analysed the developmental phases of individuals after birth. He noted that between the ages of five and seven, the body shapes typical of a male and a female started to differentiate (Pende, 1923: Vol. 1, 99). According to Pende, sexual instinct emerged during the phase of ‘little puberty’ (‘piccola pubertà’, occurring between the ages of four and seven), as did manifestations of ‘psychosexuality’ and the first artistic propensities. At the end of this phase, the ‘sexually neutral phase’ of life came to an end, and at this time the individual was particularly prone to ‘anomalies of growth’ (Pende, 1929: 3). But puberty was also a stage that posed particular risks (ibid.: 37). Between ages 11 and 14, an ‘elongation crises’ occurred, characterised by ‘sexual uncertainty’, during which the individual was particularly at risk of developing sexual dysfunctions (Pende, 1923: Vol. 1, 100). Between the ages of 13 and 18, the ‘pubertal crisis’ occurred, when external genitals and secondary sexual characteristics developed and menstruation began in girls (ibid.: 101). The ‘youth phase’, when sexual maturity was completed, took place between the ages of 17 and 21 for women and 17 and 23 for men. Between ages 24 and 40, men's and women's sexual functionality reached its peak (Pende, 1929: 1–2). If these phases characterised normal sexual development, hormonal dysfunctions might have an impact on the emotional and sexual spheres, causing abnormal development of the individual (ibid.: 37).

The Biotypological Orthogenetic Institute monitored the growth of individuals with anomalies and collected information about the Italian population. To this end, Pende developed the biotypological orthogenetic file, or ‘biotypological card’, a document adopted by eugenicists in Argentina, Brazil, and Mexico in the 1930s. In Italy, doctors completed this record of an individual's health, morphology, psychology, and general behaviour. Pende recommended that each person see a biotypological doctor every six months before they reached adulthood, and less frequently afterwards. The biotypological card contained structured headings related to the individual's genealogy; for example, the doctor had to ask whether the patient's parents and close relatives had suffered from any hereditary diseases or anomalies. Doctors identified which biotype the patient belonged to and recorded a long list of measurements such as height, weight, and size of the thorax. The card also contained sections on eccentric or unusual behaviour, sexual instinct, dietary habits, and the intellectual and moral sphere and psychological attitudes of the individual.

The basis of the biotypological card was outlined in Pende's *Endocrinologia*, which included a template that aimed to guide endocrinologists in completing endocrinological tests (Pende, 1923: Vol. 2, 706–10). The card offers an insight into the extent to which Pende instructed other endocrinologists to pay attention to the development of the genitals and the secondary sexual characteristics. For example, they were expected to record the size of the penis, the presence of facial hair, eyebrow shape, the presence and distribution of pubic hair, the timbre of the voice, the sexual instinct and vigour, fertility, when the onset of puberty occurred, signs of bodily and psychological ‘feminilism’, and the development of the chest, in particular any signs of gynaecomastia (ibid.: Vol. 2, 706–7). In a woman, ‘sexual development’ was indicated by the functioning of the internal reproductive system, the appearance of external genitals, the shape of the pelvis, breast size, the presence of pubic hair, the menstrual cycle, the sexual instinct and ‘psychosexuality’, the presence of signs of ‘masculinism’, when puberty began, signs of menopause, and any symptoms that accompanied pregnancies (ibid.: Vol. 2, 707). This inspection was complemented by a blood test and an examination of the individual’s muscle tone (ibid.: Vol. 2, 708–9). In the examination of the individual's ‘sexual development’, Pende highlighted that it was critical to carry out not only a ‘morphological sexual inspection’, but also a study of how the genitals functioned and the person's psychosexuality (ibid.: Vol. 2, 733). Important indications of endocrinological dysfunctions were female psychological characteristics in men and male psychological characteristics in women, which could exist despite the presence of normal genitals (ibid.: Vol. 2, 735).

One of the main functions of the Biotypological Orthogenetic Institute was to ‘correct’ purported ‘sexual anomalies’ and to forestall ‘moral deviations’, especially in adolescents (Barbara and Vidoni, 1933). The painstaking analysis of the individual's body in each phase of growth represented the first essential step in the elimination of hereditary weaknesses, the correction of abnormalities, and ultimately the enhancement of the Italian stock. Rectifying perceived deviations meant improving the entire population. From 1930 onwards, all members of Opera Nazionale Balilla (the Fascist youth organisation) were obliged to have a biotypological card, and in 1936, all Italian schools adopted the biotypological card as a method of recording information obtained as part of regular student health examinations (Beccalossi, 2020: 81–2).

After anomalies were identified, it was believed, it would be possible to ‘normalise’ an individual through hormone treatments. While biotypologists were uninterested in the autobiographical clinical cases of psychiatrists, the sexual knowledge resulting from biotypology expanded the taxonomy available to them, a taxonomy now based on body measurements and experimental and laboratory methods. More importantly, it had new clinical applications that seemed to have tangible effects. Doctors at the Biotypological Orthogenetic Institute adopted a range of hormone treatments, from the natural to the invasive, including surgical interventions. They administered natural therapies such as sunlight, mountain air, and mineral bath treatments, and provided guidance on special diets. These treatments aimed to stimulate the natural production of hormones in men and women. Doctors also developed a range of different radiation therapies, such as ultraviolet radiation, X-ray, phototherapy, and Marconi therapy.^9^ Like the natural therapies, these artificial therapies allegedly stimulated the production of hormones, treated certain endocrinological dysfunctions, or improved the individual's constitution (Gualco and Nardi, 1941: 147–8). Some of these treatments were designed specifically for children; for example, ultraviolet light therapy was offered to children whose bodies were smaller than normal for their age (ibid.: 146–7). Other therapies were designed to correct sexual anomalies. For instance, researchers at the Biotypological Orthogenetic Institute believed that cryptorchidism could be treated through the application of (unspecified) ‘radiation’ to the thymus gland (ibid.: 158–9).^10^ Researchers at the institute also administered a range of endocrinological therapies, such as opotherapy and glandular implants, which were used to treat infertility in men and women and impotence in men, and to normalise individuals who presented ambiguous genitals and/or divergent secondary sexual characteristics. This normalisation of individuals who presented bodily characteristics that did not conform to the perceived standards of maleness and femaleness ideally needed to start when they were still very young (ibid.: 146).

## Argentina: Correcting intersex variations

Hormone treatments such as those carried out at the Italian Biotypological Orthogenetic Institute were consolidated at a time of perceived demographic crisis. Concerns over the fate of the nation, the declining and ageing population, women's reluctance to bear children, and the decreasing virility and potency of men all lent credence to the ameliorative power of hormone treatments. Such concerns and attempts to normalise individuals could also be found in Latin American countries such as Argentina and Brazil. There are several parallels between Italy, Argentina, and Brazil. As in Southern Europe, eugenics in Latin America developed coincidentally with the resurgence of various nationalisms and the emergence of populist movements. As Finchelstein has shown, Latin America appropriated European fascism and reinvented it (Finchelstein, 2010; Pandolfi, 1999). To tackle the demographic question, the Argentinian and Brazilian governments introduced a number of public health reforms, which included maternal and child protection in the 1930s, just as the Italian Fascist regime had done. As with Italy, Argentinian and Brazilian scientists promoted this ‘positive eugenics’, encouraging optimal levels of reproduction rather than attempting to prevent those considered unfit from breeding. However, they also amplified the focus on women's reproductive and sexual roles. Within the scientific communities of Latin America, there existed a complex scientific-institutional network that became closely associated with Italian biotypology and allowed it to develop. As Nancy Stepan explores in her pioneering work on the subject, biotypological thinking and practices were adopted in countries such as Argentina, Brazil, and Mexico (Stepan, 1991), with Argentinian and Brazilian medical doctors and eugenicists who adopted biotypology cultivating an active mutual exchange of ideas and practices with their Italian colleagues (Miranda and Vallejo, 2012; Vimieiro Gomes, 2017).

In Argentina, biotypology flourished thanks to the organisational abilities of Arturo Rossi, a physician who had been the medical clinical director and taught in the Faculty of Medical Sciences at the University of Buenos Aires. He also founded the Instituto de Biotipología, Eugenesia y Medicina Social in Buenos Aires in 1931 (Rossi, 1944: Vol. 1, 39, n. 1). That same year, he travelled to Italy and spent 13 months, mainly in Genoa, studying with Pende at the Biotypological Orthogenetic Institute (Vallejo and Miranda, 2011: 63).^11^ Upon his return to Buenos Aires in February 1932, in partnership with Donato Boccia and Octavio López, Rossi founded another organisation, the Asociación Argentina de Biotipología, Eugenesia y Medicina Social (Rossi, 1944: Vol. 1, 16). In 1933, together with López and Gonzalo Bosch, Rossi also launched a fortnightly journal, the *Anales de Biotipología, Eugenesia y Medicina Social*. The *Anales* acted as a conduit for the exchange of biotypological ideas between the Northern and Southern hemispheres, and both Pende and Marañón regularly contributed articles. In 1934, the association founded the Escuela Politécnica de Biotipología, Eugenesia y Medicina Social, where hundreds of students were trained in the discipline of biotypology. Finally, in 1943, a chair of biotypology was created at the University of Buenos Aires thanks to Dr Guillermo Rothe, the Argentinian minister of justice and public education (ibid.: Vol. 1, 17; Reggiani, 2010).

As with Italian biotypology, the study of the early phases of childhood was central to Argentinian biotypology. The Asociación Argentina de Biotipología, Eugenesia y Medicina Social created a specific department, that of Eugenesia, Maternidad e Infancia, devoted to studying and caring for mothers and children. Headed by Prof. Dr Josué A. Beruti, this department was in contact with international eugenics organisations that prioritised motherhood and childhood, including the Federación latina de sociedades de eugenesia, which had its headquarters in Rome, and the Società italiana di genetica e eugenetica (Beruti, 1934). Endocrinology and biotypology provided much of the scientific rationale for the study and optimisation of the Argentinian population's potential fertility, and their analytical frameworks were used to assess which female biotypes were more fertile (Eraso, 2007).

While Rossi's contribution to the institutionalisation of Argentinian biotypology is well known (Vallejo, 2004), his work on sexual development and hormones has been overlooked. In 1944, Rossi published a three-volume work entitled *Tratado teorico pratico de biotipología y ortogenesis*, outlining his synthesis of contemporary biotypological studies. Following Pende, Rossi covered the developmental phases of men and women, paying particular attention to childhood and orthogenesis (Rossi, 1944: Vol. 1, 263–371). He also expounded the earliest phases of sexual development and the theory of latent organic bisexuality. Like Pende, Rossi believed that humans were originally bisexual and provided two pieces of scientific proof, along with cultural evidence. The first came from histological evidence, whereby the same interstitial cells could be found in both the testes and the ovaries. The second came from endocrinological evidence, with Rossi pointing out that both male and female hormones were present in men's and women's urine (ibid.: Vol. 1, 277). He argued that, humans being originally neutral, neither men nor women were completely male or female in their anatomical, functional, and psychological characteristics (ibid.: Vol. 1, 278). Finally, he emphasised that human history was full of ‘intermediate people’ (ibid.: Vol. 1, 277).

Rossi's acceptance of the theory of latent organic bisexuality and his awareness of the existence of ‘intermediate people’ throughout history did not mean greater acceptance of these phenomena. Indeed, he recommended that people working in education be aware of the original bisexuality of human beings and that when teaching, educators should exalt masculinity in boys and femininity in girls. According to Rossi, a lack of proper sexual education could cause homosexuality, which he considered a sickness and a more advanced degree of ‘intersexualism’ (Rossi, 1944: Vol. 1, 279–80). Same-sex desires had a biological origin, but they also needed external and environmental input to become manifest (ibid.: Vol. 1, 280–1). Rossi explained that both the psychoanalyst and the embryologist were aware that many ‘sexual deviations’ were a result of childhood experiences (ibid.: 287). In the prevention of homosexuality, as in that of other forms of ‘deviance’, biotypology's role was important: this discipline analysed the hereditary factors affecting an individual's development, thereby allowing eugenics programmes to improve both the individual and the race (ibid.: 294). According to Rossi, the science of orthogenesis, established by Pende, aimed to ‘normalise’ the individual and was essential for ‘human reclamation’. It studied bodily, intellectual, and moral development and their deviations. Orthogenesis served the financial, military, reproductive, and ‘spiritual’ (meaning cultural) power of a nation, but it was also different from eugenics, as it was based on more Christian, human, and scientific criteria. It was a post-natal intervention (ibid.: 294–5).

Although well versed in hormone research, Rossi was not an endocrinologist, and mainly sought to expound biotypology theories. Yet hormone treatments to normalise people who did not conform to expected standards were carried out in Argentina as well as in Italy. Enrique B. del Castillo, professor at the Institute of Physiology and director of the Department of Endocrinological Disorders at the Hospital Rivadavia in Buenos Aires, explained how in Argentina hormones were used in a practical fashion to normalise people who did not conform to perceived standards of masculinity and femininity. In 1944, the same year that Rossi published his *Treatise* on biotypology, del Castillo published *Endocrinologia clinica*. Overlooked by historians, this interesting book surveyed the ‘intersexual conditions’, their physical characteristics, and the ways in which they could be normalised.

Del Castillo explained that an individual's sex was formed in two phases: the first, when the egg was fertilised, was when the gonads of a specific sex started to develop; and in the second, when the gonads were constituted, these secreted hormones that were transported into the blood and ensured that the organism acquired the secondary sexual characters of a specific sex (del Castillo *et al.*, 1944: 490). The action of the ‘sex hormones’, a product of the genetic determination of the sex chromosomes, was extremely important in the study of sex in human beings. Following Goldschmidt, del Castillo explained that, broadly speaking, ‘intersexual’ indicated a ‘kind of sexuality that is neither male or female, but something in between the two sexes’. ‘Hermaphroditism’, meanwhile, was a general term that indicated the presence of both sexual cells (female and male) in the same organism (ibid.: 491). As historians such as Pablo Ben have shown, ideas about fluidity were present in Argentinian medicine before the rise of endocrinology (Ben, 2000a, 2000b). Indeed, del Castillo's reflections on sexual development were in line with the 19th-century European medical literature analysed above. However, endocrinology provided new clinical interventions, as del Castillo illustrated when talking about intersex variations.

Del Castillo classified several different kinds of intersex variations. These included ‘hermaphrodites’, who could be either ‘true hermaphrodites’ or ‘false hermaphrodites’, that is, individuals who had well-defined primary sexual characteristics, either ovaries or testes, but who at some point in their sexual development had their secondary sexual characteristics changed into those of the opposite sex. These were the ‘virilised woman’ and the ‘feminilised man’. Such individuals presented ‘minimal degrees of intersexuality’, such as men affected by gynaecomastia or hypospadias and male and female homosexuals. In line with Marañón, therefore, del Castillo classified homosexuality as a form of intersexuality, although he acknowledged that there was a medical debate about whether homosexuality did indeed belong to the same category of intersexuality (del Castillo *et al.*, 1944: 493).

According to del Castillo, it was impossible to provide an exhaustive overview of treatments for individuals with intersex variations, as within this category the cases were so different that each required different treatment (del Castillo *et al.*, 1944: 497). He explained that he did offer some of the available treatments to people whose bodies did not conform to accepted standards of maleness and femaleness. Female ‘pseudohermaphroditism’ was, according to del Castillo, a condition in which an individual had female gonads (ovaries), but their external genitalia had a combination of male and female characteristics. The genital ‘anomalies’ could be so pronounced and the secondary sexual characteristics so typically male that it was sometimes necessary to extract a tissue sample from the gonad and to carry out a histological examination to determine the individual’s ‘real sex’ (ibid.: 343). It did not matter whether an individual was healthy and reconciled to their condition; the doctor prescribed treatment regardless. This consisted of the extirpation of the clitoris, which in cases of female pseudohermaphroditism was hypertrophied, and a vaginal operation to shape the external genitalia into a ‘female shape’ (ibid.: 346). The operation was followed by the administration of oestrogens to stimulate the secondary sexual characteristics in both anatomical and functional terms. Del Castillo also studied and treated other sexual variations. In his taxonomy, a ‘eunuco’ was an individual whose testicular function had been lost because of castration before puberty or during adolescence. A ‘eunucoid’ had a similar condition to that of the eunuco, except that the lost testicular function was not caused by a traumatic event or surgical intervention (ibid.: 445). In either case, there was a general tendency for the individual to become taller than average (ibid.: 447). In cases of ‘eunuquismo’ and ‘eunucoidismo’, treatment was aimed at ‘normalising’ the secondary sexual characteristics and reproductive organs, and stimulating ‘spermatogenesis’ (ibid.: 453). In cases involving ‘testicular hypogonadism’, the administration of testosterone was advised.

In the first half of the 1940s, the most common way to administer testosterone in Argentina was via injection, but oral administration and grafts in the inguinal area were also possible. As del Castillo explained, the way in which testosterone was administered depended on a number of factors, including the individual's bodily response, financial circumstances, occupation, and willingness to continue the treatment (del Castillo *et al.*, 1944: 454). To treat ‘eunuquismo’ and ‘eunucoidismo’, an endocrinologist had to administer between 50 and 150 mg of testosterone propionate a week, and this needed to be given in multiple doses, possibly even on a daily basis. Grafting or implanting testosterone crystals under the skin was seen as a simple procedure, allowing for a continuous dose of testosterone, which also saved on costs and was five times stronger than injecting testosterone. For example, a graft of five compressed crystals of 200 mg each lasted for several months. Testosterone could also be administered daily in the form of a gel, which the skin absorbed, although this method was not considered satisfactory at the time (ibid.: 455).

In the interwar period, a number of clinical cases of people with intersex variations treated with hormone treatments were published in the Argentinian medical literature, each drawing on biotypological thinking to a different extent.^12^ Most of these cases focused on the body, and although endocrinological treatises debated whether homosexuality fell within the category of intersexuality, broadly speaking, the Argentinian clinical cases of intersex variations that I have analysed in my broader research did not cover the sexual instinct extensively. From opotherapy to the grafting of animal endocrine glands, hormone treatments were considered an effective way to therapeutically manipulate the body, restoring order and bringing individuals closer to the standard of the male or female sex.

## Brazil: Correcting same-sex behaviours

In Brazil, biotypology was not as popular as it was in Argentina, but it was nevertheless adopted in medical circles and taught in medical schools, and several publications were devoted to the subject. Brazilian biotypologists were eclectic and selected the most important theories and techniques for corporeal classification from both Europe and the US, adapting and reinterpreting such theories to suit local needs. Biotypology in Brazil grew more popular in scientific circles and gained institutional recognition in the 1930s, when Getúlio Vargas came to power. Historians have highlighted that the Vargas dictatorship cannot be reduced to a local version of European fascism. As Vimieiro Gomes has pointed out, the Vargas dictatorship was a ‘paradoxical mixture of regressive and progressive ideals. Alongside repressive and authoritarian measures, several economic, legislative and cultural innovations were implemented in this period’, including centralised social policies of health care and education, welfare and labour legislation, and maternal and child protection. ‘The regime also aimed to forge a nationalist ideology through a heightened sense of “Brazilianness”’ and investing in a cultural programme inspired by popular and Afro-Brazilian traditions (Vimieiro Gomes, 2017: 142; see also da Cunha, 1999). This characteristic distinguished Brazil's approach to race from Argentina's, which did not even attempt to value Afro-Argentinian traditions.

Italian biotypologists provided the main theoretical framework for Brazilian biotypologists and were held in particularly high regard (Vimieiro Gomes and Dos Santos Silva, 2019: 85). Significantly, as Vimieiro Gomes and Dos Santos Silva have shown, Italian biotypological classifications were seen as ‘an alternative to traditional racial typologies’ (ibid.: 82). Rio de Janeiro in particular became a key centre for biotypological studies. Juvenil Rocha Vaz and Waldemar Berardinelli, both professors at the Faculty of Medicine at the University of Rio de Janeiro, established and led an important Brazilian school of constitutional medicine and biotypology (Rossi, 1944: Vol. 1, 38; Vimieiro Gomes and Dos Santos Silva, 2019: 82). In the early 1930s, the University of Rio de Janeiro also established a laboratory of biotypology that was attached to the compulsory clinical courses for first-year students (Vimieiro Gomes, 2017: 147). This meant that all medical students graduating from the university were familiar with biotypology.

A number of Brazilian sexologists embraced biotypology and were concerned with ‘intermediate conditions’. Similar discourses to those found in Italy, Spain, and Argentina, linking endocrinology, biotypology, and use of hormone treatments, can be found in Brazilian medical discussions of sexuality. There was also a remarkable medical literature in Brazil that studied homosexuality within a biotypological framework, and which testifies to the use of hormone treatments to allegedly ‘cure’ same-sex desires. As in Argentina, homosexuality was considered a manifestation of intersexuality in Brazil, one that could be explained through the theory of latent organic bisexuality. But hormone research and the biotypological studies that shaped Brazilian sexology were also intertwined with criminal anthropology. This trend is best represented by the legal doctor Leonídio Ribeiro, who published the influential work on male homosexuality *Homossexualismo e endocrinologia*, and was an advocate for the ‘correction’ of the homosexual instinct through the use of hormone treatments (Green, 1999: 70–4, 110–27). In 1931, Ribeiro, who came from the Bahian School, which specialised in Afro-Brazilian studies, racial problems, and criminology, was invited to become the director of the Instituto de Identificação da Polícia Civil do Distrito Federal on the recommendation of Batista Luzardo, the chief of police. The institute's role was to create a ‘scientific police’ in Brazil and to train policemen in the use of biotypology to identify criminals. A few years later, in 1933, Ribeiro established the Laboratório de Antropologia Criminal, bringing together a team of doctors linked to the constitutional and biotypological school of Rocha Vaz, comprising Waldemar Berardinelli, Manoel Roiter, Arthur Ramos, and João Mendonça, to work in the laboratory. For Ribeiro, biotypology was a new stage in criminal anthropology, uniting biological, psychological, sociological, and endocrinological knowledge, and contributing to social politics.

The Laboratório de Antropologia Criminal, attached to the Instituto de Identificação da Polícia Civil of Rio de Janeiro, was a significant centre where research that associated homosexuality with dysfunctions of the internal secretion glands was conducted. At the laboratory, Ribeiro and Berardinelli studied male sex workers whom the police had identified as homosexuals (Berardinelli, 1936: 473–4; 1942: 610–14; Ribeiro, 1938: 104–5).^13^ Ribeiro studied 195 men arrested in Rio de Janeiro in 1932 using Cesare Lombroso's criminal anthropology and Pende's biotypology, employing anthropometric and biotypological techniques to measure their body parts, with the aim of proving the link between hormonal imbalances and homosexuality. The preliminary results of this massive study were published in the *Archivos de Medicina Legal e de Identificação*, and received the Lombroso Prize in Italy in 1933.^14^

A more complete study of these homosexuals was published under the title of *Homossexualismo e endocrinologia* in 1938, one year after Vargas had established his dictatorship in Brazil. This book became extremely influential, not only in Brazil, but also throughout Latin America and Southern Europe. It was translated into a number of other languages, with the Spanish version appearing in 1938, and the Italian version going through two editions (1939, 1940) within a couple of years. The book's influence in and beyond Brazil has resulted in extensive analysis (Green, 1999; Gutman, 2010; de Oliveira Júnior, 2012: 265–83), although the content dealing with sexual development has perhaps been less thoroughly studied. The book focuses on the 195 ‘passive pederasts’ imprisoned by the Polícia Civil Carioca, led by Dulcídio Gonçalves, who was known for his persecution of homosexuals, samba, and people of African origin. Although Ribeiro believed that personal experiences and environment could profoundly influence individual psychology and lead to homosexuality, he pinpointed the original source of sexuality in the body. Strongly influenced by endocrinological research, he viewed sexuality as a matter of hormones and chemicals (Ford, 1995: 36–7).

The preface to Ribeiro's book was written by Marañón, who rightly identified the influences of his own study, *La evolución de la sexualidad*, on Ribeiro's work. Marañón highlighted the fact that Ribeiro did not consider homosexuality a crime, but a medical ‘abnormality’, although homosexuals could become criminals should their environment lead them to commit crimes (Ribeiro, 1938: 9). Ribeiro adopted a view that was well established in Southern Europe and Latin America, Marañón explained, to the effect that homosexuality had its organic origin in the condition of intersexuality (ibid.: 10, 43). In some cases, the findings of the physical examination were self-evident; the bodies of homosexual men were thus marked by signs of intersexuality and displayed female sexual characteristics. In other cases, homosexuals' bodies appeared normal, but during puberty they began to show signs of intersexuality, such as a round-shaped body, hair distribution typical of the female sex, and gynaecomastia. Even if their body became more masculine as they grew older, their sexual inclination would remain deviant (ibid.: 10). To be complete, Ribeiro concluded, the examination of sexual inversion should include an analysis of the body's morphology during puberty, possibly with photographic records (ibid.).

In *Homossexualismo e endocrinologia*, Ribeiro discussed different interpretations of homosexuality, from the psychological to the endocrinological. He believed that although some of the arguments about homosexuality put forward by psychoanalysis were ‘acceptable’ to a certain extent, scientists were getting closer to finding a solution to the ‘problem’ of the ‘pathological deviations of human sexuality’ thanks to the ‘science of the constitution’, meaning biotypology (Ribeiro, 1938: 36–7). Ribeiro used the theory of latent organic bisexuality to explain the phenomenon of homosexuality. Making clear his own intellectual inspirations, he surveyed medical literature on the topic, starting with Gley's embryological research published in the *Revue philosophique*. He also offered a review of Kiernan's psychiatric studies on homosexuality, published in the 1880s, which had adopted the theory of latent organic bisexuality (ibid.: 37). Ribeiro employed these studies to show that homosexuals presented some characteristics of the opposite sex. In addition, he asserted, experimental endocrinological studies had confirmed late 19th-century embryological theories. Studies by Steinach in the 1910s had demonstrated that it was possible to provoke ‘hermaphroditism’ with the development of the sexual glands of the male and female sex within a single animal (ibid.: 38).

In his book, Ribeiro selected theories in an eclectic way to develop a coherent theory on the origin of homosexuality. An example of his eclectic style is his selective use of psychology and psychiatry, along with recent endocrinological explanations. Ribeiro did not entirely reject Freud's view on homosexuality. However, he looked to endocrinology to offer the definitive explanation of same-sex desires. It is in the context of using endocrinological research to explore the origin of homosexuality that Ribeiro addressed the issue of sexual development in men and women. Once again, endocrinological studies of sexual development were central to challenging the idea of two distinct sexes. Fundamentally, the Brazilian criminal anthropologist adopted the endocrinological theories of Marañón and Pende to support his views on homosexuality. First, to explain the sexual development of the individual, Ribeiro took up Marañón's endocrinological constitutional theory, which suggested that for women, for example, a sexually undifferentiated childhood was followed by a female phase typical of puberty. This phase lasted for a woman's entire sexual life, ending only with the onset of menopause, when a male phase began (Ribeiro, 1938: 42–3). In men, the female phase ended much earlier, after puberty, and was followed by a male phase. Following Marañón, Ribeiro argued that the differences between men and women depended on quantitative – not qualitative – differences in sexual characteristics (ibid.: 43). No ideal male or ideal female existed, only multiple gradations between two opposite and ideal sexual types (ibid.: 41). Second, Ribeiro reported that Pende had shown that the sexual development of an individual was determined not exclusively by the internal secretions of the genital glands, but also by those from other glands that produced a variety of hormones, all of which worked together in a complex system (ibid.: 39). As Ribeiro explained, Pende had argued that the ‘somatic and psychological syndrome’ of homosexuals could not be reduced to the genital glands alone. Pende had stressed the importance of the pathological hyperfunction of the thymus for the development of homosexuality after puberty. But the complex interaction of all hormones could help to bring about homosexuality if dysfunction occurred (ibid.: 49–50). Finally, Ribeiro firmly stated that given the current state of scientific knowledge, there was unanimity in the scientific world on the point that homosexuality was a phenomenon influenced by a state of original organic bisexuality. The two sexes could coexist in the same subject as a person passed from the neutral to the female and male phases. Moreover, it had also been proven that the two ‘sex hormones’ (male and female) had a similar chemical composition (ibid.: 43–4).

Ribeiro also devoted an entire chapter to the psychological explanation for homosexuality (Ribeiro, 1938: 147–68). Combining Krafft-Ebing's analysis with that of Marañón, he analysed the psychological characteristics of male homosexuals. Repeating late 19th-century psychiatric tenets, Ribeiro affirmed that homosexuals manifested their inclinations and typically female attitudes during childhood. For example, they preferred to play with girls, some feeling themselves to be women (ibid.: 151–2), while certain male homosexuals also enjoyed wearing women's clothes (ibid.: 154). Conflating sexual orientation and gender identity, Ribeiro, like many other sexologists of his time, confused homosexuals with transgender people.

Ribeiro explained that once sexologists had shown that homosexuality was in most cases caused by changes in the endocrine glands, it had become possible to treat it (Ribeiro, 1938: 169). He then reported on a number of experimental endocrinological treatments on homosexuals, including a pioneering experiment performed by the Viennese surgeon Robert Lichtenstern, who undertook one of the first documented testicular transplants aimed at modifying the sexual instinct of a man who was attracted to other men (ibid.: 170). Ribeiro also alluded to the famous monkey testicle grafts that Serge Voronoff had used in the 1920s to treat homosexuality (ibid.: 55, 171). He highlighted that it was very important to start hormone treatments at an early age, meaning at the beginning of puberty, to achieve better results in the treatment of homosexuals (ibid.: 172).

While Ribeiro did not mention any hormone treatment he had personally performed on homosexuals, and I have not been able to find any evidence of him trying to cure homosexuals with hormone treatments, Brazilian sexologists did offer hormone treatments to homosexuals and adopted biotypology to classify individuals. The famous sexologist Hernani de Irajá provides some evidence of how Brazilian doctors employed hormone therapies to normalise people and their sexual desires. After obtaining a degree in medicine, Irajá moved to Rio de Janeiro, where he established a private sexology practice and published sexological writings, many of which targeted a popular audience. While Irajá's central contribution to Brazilian sexology has been explored (Ezabella, 2021; Russo *et al.*, 2011), historians have paid little attention to his use of biotypology and hormones to treat homosexual patients. In 1937, he published *Tratamento dos males sexuales*, where he employed biotypological categories such as the brevilineal and longilineal constitutions to describe his homosexual patients. He also openly discussed hormone treatments that he had administered to treat ‘sexual perversions’, including homosexuality. According to Irajá, anomalies of the sexual instinct could be hereditary (biological), acquired, or a combination of both. He reported that he had studied and attempted to cure 100 cases of homosexuality with hormone therapies in his career to date, 70 male and 30 female (de Irajá, 1937: 223). Hormone treatments such as injections of testicular extracts could last several months and could be combined with other therapies, ranging from psychoanalysis to applying electric galvanic stimulation to the patient's anus (ibid.: 210–11). While Irajá observed bodily changes and a general improvement in his subjects’ health, he admitted that homosexual desires did not disappear. Out of more than 80 cases, only in 12 did Irajá notice any improvement (ibid.: 216–17). Despite such a low success rate, however, he continued to administer hormone treatments in an attempt to modify his patients’ sexual behaviour.

## Conclusion

From at least the second half of the 19th century onwards, sexologists from various medical disciplines studied human sexual development in an attempt to understand adult sexuality. In the process, they looked at the earliest phases of human development and adopted embryological views. Embryologists observed that in the first weeks of human gestation, the foetus was sexually undifferentiated. These observations gave rise to the theory of a latent organic bisexuality, which posited that the foetus was originally sexually neutral and that while the individual generally developed the sexual characteristics typical of the male or female sex, the sexual characteristics of the opposite sex remained, albeit in a latent state.

Endocrinological observations on the life cycle and bodily transformations due to hormonal changes throughout an individual's life seemed to confirm embryological studies. Endocrinologists highlighted that in the early phases of human development, from birth to childhood, the secondary sexual characteristics were not developed, and that children assigned as either male or female at birth on the basis of their genitalia fundamentally had very similar bodies. The sexual characteristics generally started developing in one direction at puberty, but these characteristics could reverse at any time due to hormonal changes or hormonal dysfunction, and this was particularly evident in women after menopause, when they tended to become more masculine. Thus, hormones came to be understood as the chemical substances that regulated an individual's growth and sexual development, with sexologists interested in this area focusing primarily on the first few decades of human existence.

Sexologists in Southern Europe and Latin America trained as endocrinologists in general, and particularly those who embraced biotypology, contributed to the proliferation of medical and clinical discourses on ‘intermediate conditions’ or ‘intersexual conditions’. Under these labels, a plethora of human variations were classified. The notion of intermediate conditions was a loose umbrella concept, with sexologists often conflating individuals with intersex variations and homosexuals. Endocrinologists and biotypologists were less interested in psychological observations, and were generally content to leave the realm of psychological investigation to their psychiatric colleagues. After all, late 19th-century psychiatric studies on ‘sexual inversion’ only seemed to confirm that homosexuals had some psychological and, quite often, physical characteristics of the opposite sex. What is perhaps surprising is that, based on their studies of intermediate conditions, sexologists in the interwar period conceptualised a biomedical continuum encompassing the (ideal) male and female sexes. In these terms, endocrinological and biotypological studies were startlingly contemporary, in that they allowed sexology to contemplate multiple variations in sex characteristics and the richness of sexual variations. At the same time, the molecular style of reasoning promoted by endocrinologists and biotypologists led medical men to think that the body, with its sexual characteristics, and even sexual instinct, were malleable. If hormones controlled the sexual development and sexual instinct of an individual, then the administration of hormones could alter both. If endocrinologists and biotypologists multiplied human variations in theory, and made human variations more visible, in practice they attempted to normalise people who did not conform to accepted standards of what it was to be male and female. Acknowledging human variations did not mean valuing diversity. In this sense, interwar sexologists trained in endocrinology and biotypology were not able to escape the sexual binarism characteristic of earlier decades.

The molecular style of reasoning promoted by endocrinology and biotypology was not exclusive to Southern Europe and Latin America. In North America, for example, Louis Berman's endocrinological studies put forward ideas similar to Pende's biotypology, and German psychiatrist Ernst Kretschmer, who had studied personality traits and mental disorders in relation to different constitutions, developed a typological system whose logic resembled that of Pende's biotypology (Nordlund, 2007; Trask, 2018). However, biotypology led to the formation of a network of sexologists who had their own circuits of knowledge production and points of reference quite distinct from those more typical of Northern Europe and North America. In this Latin circuit of sexual knowledge that I have considered, there is no trace of the ‘global supremacy’ of North American or even Northern European science. While certain major figures, such as Havelock Ellis and Magnus Hirschfield, were well known and widely utilised in the sexological literature of Southern Europe and Latin America in the interwar period, in these areas scientists such as Marañón, Pende, Ribeiro, and Lipschütz were dominant. Journals such as the Argentinian *Anales* and the Latin eugenics movement, with its regular conferences, also helped shape a mode of sexological thought that was in some respects distinct from that of Northern Europe and North America. In Latin countries, hormone research and associated treatments shaped the development of sexology in the first half of the 20th century. Here, a molecular style of reasoning, which was partly linked to the past (through the humoral body) and at the same time innovative (through new clinical interventions), greatly contributed to shaping how sexologists perceived both sex and sexuality.

## Notes

1. Translations of quotations from non-English sources throughout the article are my own.

2. In this context, ‘latent organic bisexuality’ refers to the body being sexually neutral, not to sexual orientation.

3. On Darwin and bisexuality in man, see Sulloway (1979: 158–9).

4. This work was originally published in 1929 under the title *Los estados intersexuales en la especie humana*.

5. On Marañón’s theory of intersexuality, see Cleminson and Vázquez García (2009: 123–78).

6. Contemporary scientists recognised Marañón as a scientists who adopted biotypology; see, for example, the Brazilian endocrinologist Berardinelli’s *Biotipologia* (1936).

7. In this context, ‘orthogenetic’ refers to orthogenesis as a branch of constitutional medicine that dealt with issues related to individual growth; that is, with the bodily and psychological development of men and women.

8. Pende does not define ‘erotism’. It is plausible to assume he meant excessive sexual desire.

9. Marconi therapy involved exposing patients to electromagnetic waves.

10. Cryptorchidism is a condition in which one or both testes fail to descend from the abdomen into the scrotum and the individual’s a body fat distribution resembles that of a girl.

11. The Ministry of Foreign Affairs and Worship (Ministerio de Relaciones Exteriores y Culto) is the government ministry that oversees the foreign relations of Argentina.

12. See, e.g., Castex and Camauër (1927a, 1927b); Castex and Waldrorp (1920).

13. Male homosexuality was not illegal in Brazil but was actively persecuted by the police.

14. The Lombroso Prize was awarded in Italy to the best international study on criminal anthropology. It was established in honour of the criminal anthropologist Cesare Lombroso.

## References

[bibr1-09526951231213028] Angelides S. (2001) A History of Bisexuality. Chicago, IL: University of Chicago Press.

[bibr2-09526951231213028] Barbara M. Vidoni G. (1933) L’Istituto Biotipologico Ortogenetico di Genova [The Biotypological Orthogenetic Institute in Genoa]. Genoa: Badiali.

[bibr3-09526951231213028] Beccalossi C. (2020) ‘Optimizing and Normalizing the Population Through Hormone Therapies in Italian Science, *c.*1926–1950’, British Journal for the History of Science 53(1): 67–88. 31933444 10.1017/S0007087419000906

[bibr5-09526951231213028] Ben P. (2000a) ‘Cuerpos femininos y cuerpos abyectos. La construcción anamatómica de la femininad en la medicine argentina’ [Feminine Bodies and Abject Bodies: The Anatomical Construction of Femininity in Argentinian Medicine], in Lozano F. G. Pita V. S. Ini M. G. (eds) Historia de las mujeres en la Argentina: Vol. 1 [Women’s History in Argentina: Vol. 1]. Buenos Aires: Taurus, pp. 253–67.

[bibr4-09526951231213028] Ben P. (2000b) ‘Muestrame tus genitales y te diré quién eres. El “hermafroditismo” en la Argentina finisecular y de principios del siglo XX’ [Show Me Your Genitals and I Will Tell You Who You Are: ‘Hermaphroditism’ in *fin de siècle* Argentina and at the Beginning of the 20th Century], in Acha O. Halperín P. (eds) Cuerpos, géneros e identidades. Estudios de Historia de Género en Argentina [Bodies, Genders, and Identities: Studies in Gender History in Argentina]. Buenos Aires: Ediciones del signo, pp. 61–105.

[bibr6-09526951231213028] Berardinelli W. (1936) Biotypologia [Biotypology]. Rio de Janeiro: Alves.

[bibr7-09526951231213028] Berardinelli W. (1942) Tratado de biotipologia e patologia consititutional [Treatise of Biotypology and Constitutional Pathology]. Rio de Janeiro: Alves.

[bibr8-09526951231213028] Beruti J. A. (1934) ‘Reglamento, Organización y Plan de Acción de la Sección Eugenesia, Maternidad e Infancia de la Asociación Argentina de Biotipología, Eugenesia y Medicina Social’ [Regulations, Organisation, and Action Plan of the Eugenics, Motherhood, and Childhood Department of the Argentinian Association of Biotypology, Eugenics, and Social Medicine], Anales: Biotipologia, Eugenesia y Medicina Social 2(32): 2–3.

[bibr9-09526951231213028] Brooks R. (2012) ‘Transforming Sexuality: The Medical Sources of Karl Heinrich Ulrichs (1825–85) and the Origins of the Theory of Bisexuality’, Journal of the History of Medicine and Allied Sciences 67(2): 177–216. 21081540 10.1093/jhmas/jrq064

[bibr10-09526951231213028] Cassata F. (2006) Molti, sani, forti. L’eugenetica in Italia [Many, Healthy, and Strong: Eugenics in Italy]. Turin: Bollati Bolinghieri.

[bibr11-09526951231213028] Castex M. R. Camauër A. F. (1927a) ‘Feminilismo diencefálico’ [Diencephalic Feminilism], La prensa medica argentina 13(23): 759–73.

[bibr12-09526951231213028] Castex M. R. Camauër A. F. (1927b) ‘Feminilismo diencefálico. Conclusiones que se desprenden del studio de los casos ya publicados’ [Diencephalic Feminilism: Conclusions Emerging From the Study of the Cases Already Published], La prensa medica argentina 13(24): 790–6.

[bibr13-09526951231213028] Castex M. R. Waldrorp C. P. (1920) ‘Tres observaciones clinicas de hipogenitalismo primitivo’ [Three Clinical Observations on Primitive Hypogenitalism], Revista de la Asociación Médica Argentina 32: 280–98.

[bibr14-09526951231213028] Cepada A. Rustoyburu C. (2014) De las hormonas sexuadas al Viagra. Ciencia, Medicina e Sexualidad en Argentina y Brasil [From Sex Hormones to Viagra: Science, Medicine, and Sexuality in Argentina and Brazil]. Mar del Plata: Eudem.

[bibr15-09526951231213028] Chauncey G. (1982–3) ‘From Sexual Inversion to Homosexuality: Medicine and the Changing Conceptualization of Female Deviance’, Salmagundi 58/9: 114–46.

[bibr16-09526951231213028] Clarke A. E. (1998) Disciplining Reproduction: Modernity, American Life Sciences and the Problems of Sex. Berkeley, CA: University of California Press.

[bibr17-09526951231213028] Cleminson R. (2000) Anarchism, Science and Sex: Eugenics in Eastern Spain, 1900–1937. Oxford: Peter Lang.

[bibr18-09526951231213028] Cleminson R. Vázquez García F. (2007) ‘Los Invisibles’: A History of Male Homosexuality in Spain, 1850–1939. Cardiff: University of Wales Press.

[bibr19-09526951231213028] Cleminson R. Vázquez García F. (2009) Hermaphroditism, Medical Science and Sexual Identity in Spain, 1850–1960. Cardiff: University of Wales Press.

[bibr20-09526951231213028] da Cunha O. M. G. (1999) ‘Your Soul in Your Palm: Identifying the Race and Inventing the Nation’, in Pandolfi D. (ed.) Repensando o Estado Novo [Rethinking the Estado Novo]. Rio de Janeiro: Fundação Getulio Vergas, pp. 257–88.

[bibr21-09526951231213028] Darwin C. (1868) Variations of Animals and Plants Under Domestication: Vol. 2. London: John Murray.

[bibr22-09526951231213028] Davidson A. I. (2001) The Emergence of Sexuality: Historical Epistemology and the Formation of Concepts. Cambridge, MA: Harvard University Press.

[bibr23-09526951231213028] de Irajá H. (1937) Tratamento dos males sexuaes [Treatment of Sexual Illnesses] (2nd ed.). Rio de Janeiro: Freitas Bastos.

[bibr24-09526951231213028] del Castillo E. B. Reforzo Membrives J. de la Balze F. A. Galli Maininim C. (1944) Endocrinologia clinica [Clinical Endocrinology]. Buenos Aires: El Ateneo.

[bibr25-09526951231213028] de Oliveira Júnior A. (2012) ‘De monstris a anormais: a construçao da endocrinologia criminal no Brasil, 1930 a 1950’ [From Monsters to Abnormals: The Construction of Endocrinology in Brazil, 1930–50], PhD thesis, Universidade Federal de Santa Catarina.

[bibr26-09526951231213028] Ellis H. (1927) Studies in the Psychology of Sex: Vol. 2. Sexual Inversion. Philadelphia, PA: Davis.

[bibr27-09526951231213028] Eraso Y. (2007) ‘Biotypology, Endocrinology, and Sterilization: The Practice of Eugenics in the Treatment of Argentinian Women During the 1930s’, Bulletin of the History of Medicine 81(4): 793–822. 18084107 10.1353/bhm.2007.0130 PMC2629848

[bibr28-09526951231213028] Ezabella A. (2021) ‘Hernani de Irajá and the Early Years of Brazilian Sexology’, in Giami A. Levinson S. (eds) Histories of Sexology: Between Science and Politics. Cham: Palgrave Macmillan, pp. 225–43.

[bibr29-09526951231213028] Fausto-Sterling A. (2000) Sexing the Body: Gender Politics and the Construction of Sexuality. New York, NY: Basic Books.

[bibr30-09526951231213028] Ferla L. (2004) ‘Gregorio Marañón y la apropriación de la homosexualidad por la medicina legal brasileña’ [Gregorio Marañón and the Appropriation of Homosexuality by Brazilian Legal Medicine], Frenia 4(1): 53–76.

[bibr31-09526951231213028] Finchelstein F. (2010) Transatlantic Fascism: Ideology, Violence, and the Sacred in Argentina and Italy, 1919–1945. Durham, NC: Duke University Press.

[bibr32-09526951231213028] Ford T. (1995) ‘Passion in the Eye of the Beholder: Sexuality as Seen by Brazilian Sexologists, 1900–1940’, PhD thesis, Vanderbilt University.

[bibr33-09526951231213028] Gaudillière J. P. (2003) ‘La fabrique moléculaire du genre: Hormones sexuelles, industrie et médecine avant la pilule’ [The Molecular Factory of Gender: Sex Hormones, Industry, and Medicine Before the Pill], Cahiers du genre 1(34): 57–80.

[bibr34-09526951231213028] Gaudillière J. P. (2004) ‘Genesis and Development of a Biomedical Object: Style of Thought, Style of Work and the History of Sex Steroids’, Studies in History and Philosophy of Science Part C: Studies in History and Philosophy of Biological and Biomedical Sciences 35(3): 525–43.

[bibr35-09526951231213028] Gley E. (1884) ‘Les aberrations de l’instinct sexuel d’après des travaux récents’ [The Aberration of the Sexual Instinct According to Recent Work], Revue philosophique 17: 66–92.

[bibr36-09526951231213028] Goldschmidt R. (1916) ‘Experimental Intersexuality and the Sex-Problem’, American Naturalist 50(600): 705–18.

[bibr37-09526951231213028] Goldschmidt R. (1917) ‘Intersexuality and the Endocrine Aspect of Sex’, Endocrinology 1(4): 433–56.

[bibr38-09526951231213028] Green J. N. (1999) Beyond Carnival: Male Homosexuality in Twentieth-Century Brazil. Chicago, IL: University of Chicago Press.

[bibr39-09526951231213028] Gualco S. Nardi A. (1941) L’Istituto Biotipologico Ortogenetico di Roma [The Biotypological Orthogenetic Institute in Rome]. Rome: Proja.

[bibr40-09526951231213028] Gutman G. (2010) ‘Criminologia, Antropologia e Medicina Legal. Um personagem central: Leonídio Ribeiro’ [Criminology, Anthropology and Legal Medicine: A Central Figure: Leonídio Ribeiro], Revista Latinoamericana de psicopatologia fundamental 13(3): 482–97.

[bibr41-09526951231213028] Haynes D. Fuechtner V. Jones R. , eds (2017) A Global History of Sexual Science, 1880–1960. Oakland, CA: University of California Press.

[bibr42-09526951231213028] Herrn R. (1995) ‘On the History of Biological Theories of Homosexuality’, in de Cecco J. P. Parker D. A. (eds) Sex, Cells and Same-Sex Desires. New York, NY: Howarth Press, pp. 31–56. 10.1300/J082v28n01_03 7560922

[bibr43-09526951231213028] Lima R. R. (2021) ‘Hormônios, clínica e eugenia: A trajetória da organoterapia na endocrinologia brasileira (1893–1945)’ [Hormones, Clinics, and Eugenics: The Trajectory of Organotherapy in Brazilian Endocrinology, 1893–1945], PhD thesis, Casa de Oswaldo Cruz – FIOCRUZ.

[bibr44-09526951231213028] Linge I. (2021) ‘The Potency of the Butterfly: The Reception of Richard B. Goldschmidt’s Animal Experiments in German Sexology Around 1920’, History of the Human Sciences 34(1): 40–70. 33627957 10.1177/0952695119890545 PMC7863117

[bibr45-09526951231213028] MacMillan K. T. (2017) ‘“Forms So Attenuated That They Merge Into Normality Itself”: Alexander Lipschütz, Gregorio Marañón, and Theories of Intersexuality in Chile, Circa 1930’, in Haynes D. Fuechtner V. Jones R. (eds) A Global History of Sexual Science, 1880–1960. Oakland, CA: University of California Press, pp. 330–52.

[bibr46-09526951231213028] Mantovani C. (2004) Rigenerare la società. L’eugenetica in Italia dalle origini Ottocentesche agli anni Trenta [Regenerating Society: Eugenics in Italy From Its 19th-Century Origins to the 1930s]. Soveria Mannelli: Rubettino.

[bibr47-09526951231213028] Marañón G. (1930) La evolución de la sexualidad y de los estados intersexuales [The Evolution of Sex and Intersexual Conditions]. Madrid: Javier Morata.

[bibr48-09526951231213028] Marañón G. (1932) The Evolution of Sex and Intersexual Conditions, trans. Wells W. B. . London: George Allen.

[bibr49-09526951231213028] Marañón G. (1935) Ginecologia endocrina [Gynaecological Endocrinology]. Madrid: Espasa Calpe.

[bibr50-09526951231213028] Marañón G. (1972[1926]) Gorgos y flacos [Fat and Skinny], in Obras completas: Vol. 8 [Complete Works: Vol. 8]. Madrid: Espasa Calpe, pp. 372–412.

[bibr51-09526951231213028] Miranda M. (2003) ‘La antorcha de cupido: Eugenesia, biotipología y eugamia en Argentina, 1930–1970’ [Cupid’s Torch: Eugenics, Biotypology, and Eugamia in Argentina, 1930–70], Asclepio 55: 231–55.

[bibr52-09526951231213028] Miranda M. (2011) Controlar lo incotrolable. Una historia de la sexualidad en la Argentina [Controlling the Uncontrollable: A History of Sexuality in Argentina]. Buenos Aires: Biblos.

[bibr53-09526951231213028] Miranda M. Vallejo G. , eds (2012) Una historia de la eugenesia. Argentina y la redes biotipologicas internacionales, 1912–1945 [A History of Eugenics: Argentina and International Biotypological Networks, 1912–45]. Buenos Aires: Biblos.

[bibr54-09526951231213028] Nordlund C. (2007) ‘Endocrinology and Expectations in 1930s America: Louis Berman’s Ideas on New Creations in Human Beings’, British Journal for the History of Science 40(1): 83–104. 17575959 10.1017/s0007087406009113

[bibr55-09526951231213028] Oudshoorn N. (1990) ‘Endocrinologists and the Conceptualization of Sex, 1920–1940’, Journal of the History of Biology 23(2): 163–86. 11622316 10.1007/BF00141469

[bibr56-09526951231213028] Oudshoorn N. (1994) Beyond the Natural Body: An Archeology of Sex Hormones. London: Routledge.

[bibr57-09526951231213028] Pandolfi D. , ed. (1999) Repensando o Estado Novo [Rethinking the Estado Novo]. Rio de Janeiro: Fundação Getulio Vergas.

[bibr58-09526951231213028] Pende N. (1923) Endocrinologia. Patologia e clinica degli organi a secrezione interna [Endocrinology: Pathology and Clinics of the Internal Secretions Glands] (3rd ed., Vols 1–2). Milan: Vallardi.

[bibr59-09526951231213028] Pende N. (1929) Anomalie della crescenza fisica e psichica. Lavori dell’Istituto Biotipologico-Ortogenetico di Genova. Anno 1927–1928: Vol. 2 [Anomalies of Bodily and Psychic Development: The Works of the Biotypological Orthogenetic Institute in Genoa, 1927–8: Vol. 2]. Bologna: Cappelli.

[bibr60-09526951231213028] Pende N. (1933) Bonifica umana razionale e biologia politica [Rational Human Reclamation and Political Biology]. Bologna: Cappelli.

[bibr61-09526951231213028] Pende N. (1939) Trattato di biotipologia umana individuale e sociale con applicazioni alla medicina preventive, alla clinica, alla politica biologica, alla sociologia [Treatise on Individual and Social Human Biotypology, With Applications in Preventive Medicine, the Clinic, Biological Politics, and Sociology]. Milan: Vallardi.

[bibr62-09526951231213028] Preciado B. (2008) Testo yonqui [Testo Junkie]. Madrid: Espasa Calpe.

[bibr63-09526951231213028] Raj K. (2013) ‘Beyond Postcolonialism … and Postpositivism: Circulation and the Global History of Science’, Isis 104(2): 337–47. 23961692 10.1086/670951

[bibr64-09526951231213028] Reggiani A. H. (2010) ‘Depopulation, Fascism, and Eugenics in 1930s Argentina’, Hispanic American Historical Review 90(2): 283–318.

[bibr65-09526951231213028] Ribeiro L. (1938) Homossexualismo e endocrinologia [Homosexuality and Endocrinology]. Rio de Janeiro: Alves.

[bibr66-09526951231213028] Roberts C. (2007) Messangers of Sex: Hormones, Biomedicine and Feminism. Cambridge: Cambridge University Press.

[bibr67-09526951231213028] Rohden F. (2001) Uma ciência da diferença: Sexo e gênero na medicina da mulher [A Science of Difference: Sex and Gender in Women’s Medicine]. Rio de Janeiro: FIOCRUZ.

[bibr68-09526951231213028] Rohden F. (2008) ‘O império dos hormônios e a construção da diferença entre os sexos’ [The Empire of Hormones and the Construction of the Difference Between the Sexes], História, Ciências, Saúde- manguinhos 15: 133–52. 19397033 10.1590/s0104-59702008000500007

[bibr69-09526951231213028] Rose N. (2007) The Politics of Life Itself: Biomedicine, Power, and Subjectivity in the Twenty-First Century. Princeton, NJ: Princeton University Press.

[bibr70-09526951231213028] Rossi A. R. (1944) Tratado teorico pratico de biotipología y ortogenesis [Treatise on the Theory and Practice of Biotypology and Orthogenesis] (Vols 1–3). Buenos Aires: Editorial Ideas.

[bibr71-09526951231213028] Russo J. Rohden F. Torres I. Faro L. Fisher Nucci M. Giami A. (2011) Sexualidade, ciência e profissâo no Brasil [Sexuality, Science, and Profession in Brazil]. Rio de Janeiro: Clam.

[bibr72-09526951231213028] Rustoyburu C. (2012) ‘Infancia, hormones y género. Un análisis histórico de los discursos de la biotipologia en Argentina en los años 1930’ [Childhood, Hormones, and Gender: A Historical Analysis of the Biotypological Discourses in Argentina in the 1930s], Sexualidad, salud y sociedad 11: 9–36.

[bibr73-09526951231213028] Sengoopta C. (2006) The Most Secret Quintessence of Life: Sex, Glands, and Hormones, 1850–1950. Chicago, IL: University of Chicago Press.

[bibr74-09526951231213028] Stepan N. L. (1991) The Hour of Eugenics: Race, Gender and Nation in Latin America. Ithaca, NY: Cornell University Press.

[bibr75-09526951231213028] Stoler A. L. (2001) ‘Tense and Tender Ties’, Journal of American History 88(3): 829–65. 17654807

[bibr76-09526951231213028] Sulloway F. (1979) Freud, Biologist of the Mind: Beyond the Psychoanalytic Legend. New York, NY: Basic Books.

[bibr500-09526951231213028] Trask, A. (2018) ‘Remaking Men: Masculinity, Homosexuality and Constitutional Medicine in Germany, 1914-1933’, *German History* 36(2): 181–206.

[bibr77-09526951231213028] Turda M. Gillette A. (2014) Latin Eugenics in Comparative Perspective. London: Bloomsbury.

[bibr78-09526951231213028] Vallejo G. (2004) ‘El ojo del podere en el espacio del saber: Los institutos de biotipologia’, Asclepio 51(1): 219–44.

[bibr79-09526951231213028] Vallejo G. Miranda M. (2004) ‘Los saberer del poder: Eugenesia y biotipolpogia en la Argentina del siglo XX’ [The Knowledge of Power: Eugenics and Biotypology in 20th-Century Argentina], Revista de Indias 64(231): 425–44.

[bibr80-09526951231213028] Vallejo G. Miranda M. (2011) ‘“Civilizar la libido”: Estrategias ambientales de la eugenesia en la Argentina’ [Civilising the Libido: Eugenic’s Enviromental Strategies in Argentina], Iberoamericana 11(41): 57–75.

[bibr81-09526951231213028] Vimieiro Gomes A. C. (2017) ‘Science, Constitutional Medicine and National Bodily Identity in Brazilian Biotypology During the 1930s’, Social History of Medicine 30(1): 137–57.

[bibr82-09526951231213028] Vimieiro Gomes A. C. Dos Santos Silva A. L. (2019) ‘Average, Normal, and Beautiful: Representations of Bodies in Brazilian Biotypology (1930–1940)’, Journal of Iberian and Latin American Studies 25(1): 81–103.

